# A prolonged cholera outbreak caused by drinking contaminated stream water, Kyangwali refugee settlement, Hoima District, Western Uganda: 2018

**DOI:** 10.1186/s40249-020-00761-9

**Published:** 2020-11-04

**Authors:** Fred Monje, Alex Riolexus Ario, Angella Musewa, Kenneth Bainomugisha, Bernadette Basuta Mirembe, Dativa Maria Aliddeki, Daniel Eurien, Godfrey Nsereko, Carol Nanziri, Esther Kisaakye, Vivian Ntono, Benon Kwesiga, Daniel Kadobera, Lilian Bulage, Godfrey Bwire, Patrick Tusiime, Julie Harris, Bao-Ping Zhu

**Affiliations:** 1 Uganda Public Health Fellowship Program, Kampala, Uganda; 2 Ministry of Health, Kampala, Uganda; 3 Division of Global Health Protection, Center for Global Health, US Centers for Disease Control and Prevention, Atlanta, USA; 4 US Centers for Disease Control and Prevention, Kampala, Uganda

**Keywords:** Outbreak, Cholera, Refugees, Uganda

## Abstract

**Background:**

On 23 February 2018, the Uganda Ministry of Health (MOH) declared a cholera outbreak affecting more than 60 persons in Kyangwali Refugee Settlement, Hoima District, bordering the Democratic Republic of Congo (DRC). We investigated to determine the outbreak scope and risk factors for transmission, and recommend evidence-based control measures.

**Methods:**

We defined a suspected case as sudden onset of watery diarrhoea in any person aged ≥ 2 years in Hoima District, 1 February–9 May 2018. A confirmed case was a suspected case with *Vibrio cholerae* cultured from a stool sample. We found cases by active community search and record reviews at Cholera Treatment Centres. We calculated case-fatality rates (CFR) and attack rates (AR) by sub-county and nationality. In a case-control study, we compared exposure factors among case- and control-households. We estimated the association between the exposures and outcome using Mantel-Haenszel method. We conducted an environmental assessment in the refugee settlement, including testing samples of stream water, tank water, and spring water for presence of fecal coliforms. We tested suspected cholera cases using cholera rapid diagnostic test (RDT) kits followed by culture for confirmation.

**Results:**

We identified 2122 case-patients and 44 deaths (CFR = 2.1%). Case-patients originating from Demographic Republic of Congo were the most affected (AR = 15/1000). The overall attack rate in Hoima District was 3.2/1000, with Kyangwali sub-county being the most affected (AR = 13/1000). The outbreak lasted 4 months, which was a multiple point-source. Environmental assessment showed that a stream separating two villages in Kyangwali Refugee Settlement was a site of open defecation for refugees. Among three water sources tested, only stream water was feacally-contaminated, yielding > 100 CFU/100 ml. Of 130 stool samples tested, 124 (95%) yielded *V. cholerae* by culture*.* Stream water was most strongly associated with illness (odds ratio [*OR*] = 14.2, 95% *CI*: 1.5–133), although tank water also appeared to be independently associated with illness (*OR* = 11.6, 95% *CI*: 1.4–94). Persons who drank tank and stream water had a 17-fold higher odds of illness compared with persons who drank from other sources (*OR* = 17.3, 95% *CI*: 2.2–137).

**Conclusions:**

Our investigation demonstrated that this was a prolonged cholera outbreak that affected four sub-counties and two divisions in Hoima District, and was associated with drinking of contaminated stream water. In addition, tank water also appears to be unsafe. We recommended boiling drinking water, increasing latrine coverage, and provision of safe water by the District and entire High Commission for refugees.

## Background

Cholera is a severe gastrointestinal bacterial disease caused by a gram-negative, non-spore-forming curved rod known as *Vibrio cholerae* [1, 2]. Though there are over 200 sero-groups of *V. cholerae* globally, sero-groups O1 and O139 are most commonly associated with epidemics [3]*.* Serogroup O1 consists of the serotypes Ogawa, Inaba and Hikojima; O139 is not found in Africa [4]*.* Despite having proven interventions for cholera control and prevention, approximately 3 million cholera cases occur annually in endemic countries, with an estimated 95 000 deaths (case fatality rate [CFR] = 3.3%) [5]. Many of these cases occur in sub-Saharan Africa, including Uganda [6].

*Cholera is* caused by ingestion of food or water contaminated with *V. cholerae* and can result in death within hours if appropriate treatment is not administered [7]. The signs and symptoms of cholera include profuse painless watery stools, vomiting, abdominal discomfort or cramping due to fluid distention of the bowel, and, rarely, fever [8]. Cholera bacteria are passed in the stool or vomitus of infected persons, and as a result mainly affect communities with poor water and sanitation infrastructure [9]. Disease is managed with oral rehydration solution (ORS) and, in extreme cases, intravenous rehydration and antibiotics [4]. The WHO also recommends the use of cholera vaccine in combination with other preventive measures in communities at high risk [4]. A cholera rapid diagnostic test (RDT) can provide an early warning to public health officials about occurrence of cholera outbreaks; however, due to its’ relatively low specificity, it is recommended that fecal specimens that test positive for *V. cholerae* by the Crystal® VC dipstick be confirmed using culture-based methods [10, 11].

Since 1997, cholera cases have been reported annually in Uganda, including a major epidemic that occurred in 1998, with nearly 50 000 reported cases [11]. Of the estimated 11 000 cholera cases that occur in Uganda every year, it is estimated that 81% occur in a relatively small number of districts, comprising about 24% of country’s population. These include the rural areas bordering the Demographic Republic of Congo, South Sudan, and Kenya as well as the slums of Kampala City [12].

Hoima District located in Western Uganda is naturally well endowed with a rich varied landscape and ecosystems. The safe water supply coverage of Hoima District is about 74.2%, with wide disparities among subcounties [13]. The subcounties with the least safe water coverage are Buseruka (39.1%) and Kwangwali (47.4%). These subcounties are along the shores of Lake Albert with poor rock formation-hence cheap technologies like spring water protection, and shallow well construction are not applicable in some areas making safe water provision difficult. Additionally, the subcounties with the least safe water coverage also have the lowest latrine coverage in the district i.e. Buseruka (57%) and Kwangwali (59%) [13]. Accordingly, cholera is endemic in Hoima District, with outbreaks occurring every year since 2012 [14–17]. However, very few of the reported outbreaks have been epidemiologically characterized possibly because most people infected with *V. cholera* do not develop any symptoms; and among people who develop symptoms, the majority have mild or moderate symptoms [8].

On 15 February 2018, a 60-year-old man living at Sebagoro landing site, Lake Albert, Kabwoya sub-county, Hoima District presented with vomiting, fever, and acute watery diarrhoea. On the same day, three deaths occurring in the nearby Kyangwali Refugee Settlement were reported to Hoima District Health Office. The Hoima District hospital laboratory team cultured six stool samples from suspected cholera cases; three yielded *V. cholerae*. The Ministry of Health declared an outbreak of cholera in Hoima District on 23 February 2018 and sent a Rapid Response Team to support the district. We investigated to determine the outbreak scope, risk factors for transmission, and recommend evidence-based control measures.

## Methods

### Outbreak area

**Fig. 1:**
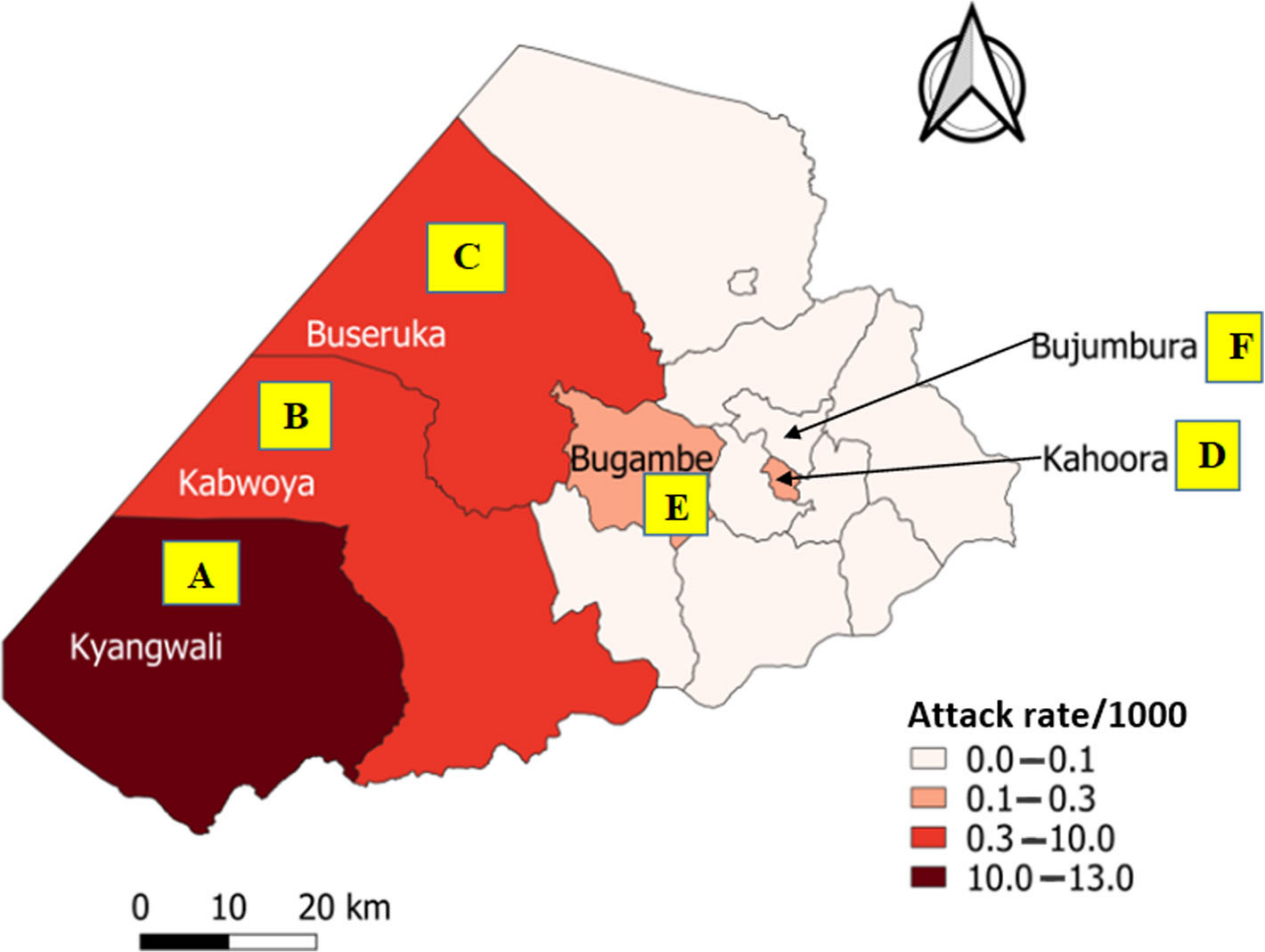
Cholera attack rates per 1000 population by sub-county Hoima District, Uganda: February–May, 2018

Hoima District is located in Western Uganda and is bordered by several districts, including the DRC across Lake Albert to the west [18]. Hoima District had a total population of 668 900 in 2018 [19]. Kyangwali Refugee Settlement accommodates close to 83 000 refugees, most from the DRC, and is located in Hoima District [20] (Fig. 1). The refugee settlement is divided into three blocks: Maratatu A, Maratatu B, and Maratatu C (Fig. 1). The refuge settlement had three main water sources, namely tank water, stream water and spring water. Tank water was the official water source supplied to the refugees in the camp. However, there were very long queues to the tank water all the time. Consequently, people resorted to alternate water sources (stream water, spring water and others) regardless of the safety of the water source.

### Case definition and case-finding

We defined a suspected case as onset of acute watery diarrhoea in any person ≥ 2 years in Hoima District from 1 February to 9 May, 2018. A confirmed case was a suspected case with *V. cholerae* isolated from stool samples by bacteriological culture. To identify cases, we reviewed medical records from two Cholera Treatment Centres (CTCs) that were about 38 km apart and conducted active community searches in the affected sub-counties to generate and update the line list. The variables captured included patient ID, gender, age, location (village, parish, sub-county), signs and symptoms, date of onset, diagnosis (clinical or lab-confirmed), nationality, refugee status, patient status (alive or dead), and drinking water sources (stream water, tank water, spring water and other sources).

### Descriptive epidemiology and hypothesis generation

For descriptive epidemiology, we considered case-patients by date of onset of signs and symptoms, by person variables (age, gender and nationality), and by sub-county. We computed attack rates by sub-county, gender, nationality, and age group (2–5, 6–17, 18–30, 31–59, 60–95). To generate the hypotheses, we interviewed 14 case-patients found in the most affected sub-county (Kyangwali) as per the descriptive epidemiology findings to identify possible risk factors associated with the outbreak.

### Case-control study

We conducted a case-control study by collecting data using a standard questionnaire with a focus on exposure factors obtained during hypothesis generation. In our case-control study, the sampling unit was a household. Thus, we defined a case-household as a household with one or more suspected cholera case-patients who sought care at CTCs or health posts in the camp from 23 February to 3 March 2018. A control-household was a household that did not have cholera patients during the outbreak period. During selection of case-households, we enrolled all case-households in the most affected blocks of Maratatu B and Maratatu C resulting into 73 case-households. Maratatu B and C had an estimated 4000 households. Using systematic random sampling, we selected every 40th unaffected household in Maratatu B and Maratatu C as control-households. In total, we selected 107 control-households.

Guided by findings from hypothesis generation interviews, we administered a questionnaire to the appropriate case-households and control-households to obtain information on their water exposures. We asked the respondents where they usually collected drinking water, treatment methods, storage and how they fetched it from storage containers. If the households never drank from the known water sources, we asked where they usually obtained drinks. We also collected information on demographic variables.

We compared exposures in 73 case-households and 107 control-households, frequency-matched by residence (village/block) in Kyangwali Refugee Settlement. We then estimated the association between the exposures and outcome using Mantel-Haenszel method. We further assessed the odds ratio (*OR*) for different combinations of the three water source types available in the settlement, to identify confounding as well as competing risk factors.

### Environmental assessments and laboratory investigations

We reviewed meteorological records at Hoima District and worldweatheronline.com to obtain rainfall data for Hoima District. We conducted an environmental assessment in the refugee settlement focusing on landscape and location of the households, water sources, and latrines. The refugee camp had three types of water sources for drinking, including stream water, spring water, and tank water. Between 23 February and 3 March 2018, we collected six water samples from different points along a stream running between villages in the refugee settlement, five water samples from water tanks in Maratatu, and five water samples from the spring water that was not a protected source (water from an underground source within Kyangwali refugee settlement) for laboratory testing for fecal contamination. We used the Most Probable Number (MPN) method using presumptive test and confirmatory test to determine the presence of fecal coliforms in the water samples [21–24].

Additionally, stool samples were collected from 15 February 2018 to 9 May 2018 from suspected case-patients in affected areas. Using swabs, stool samples were placed in Cary–Blair media, and transported within 12 h in cool boxes to the Uganda National Health Laboratory Services (UNHLS) in Kampala for testing. All samples were first tested using cholera RDT kit (Crystal® VC dipstick, Span Diagnostics Limited, Surat, India, suboptimal sensitivity and specificity), and later tested by bacteriological culture. Only samples that tested RDT-positive were retested by culture for confirmation. The swabs were first cultured in alkaline peptone water at 37 °C for 18–24 h and then sub-cultured on Thiosulphate-Citrate-Bile-Salts Sucrose (TCBS™; EIKEN Japan) agar. Upon identification, the *V. cholera* isolates were further evaluated to ascertain the serogroup and serotype by agglutination with polyvalent O1 and monospecific Ogawa and Inaba antisera [10, 25].

### Data analysis

We entered the data into Microsoft Excel version 2010 and exported it to Epi-Info version 7.2.2.6 (Centers for Disease Control and Prevention, Atlanta, Georgia, USA) for analysis. We performed descriptive analysis of the case-patients by person, place and time and summarized the independent variables as follows: signs and symptoms as frequencies, while age, gender, nationality and subcounty as attack rates. To establish potential risk factors of cholera outbreak in the refugee settlement, we dichotomized the qualitative exposures (three water sources) and compared them among case-households versus control-households using Chi-Square test at 95% confidence level (*CI*). We then estimated the association between the exposures and outcome using Mantel-Haenszel method. We further assessed the odds ratio for different combinations of the three water source types available in the settlement, to identify confounding as well as competing risk factors.

### Ethical approval and consent to participate

The investigation was authorized by Ministry of Health, Hoima District Local Government, and the Commandant of Kyangwali Refugee Settlement. This investigation was conducted in response to a public health emergency and was therefore determined by the Centers for Disease Control to be non-research. Thus, we do not need any other permission from Institutional Review Board according to the Ugandan policies and guidelines and are free to publish the work. Verbal informed consent in the local language was sought from respondents or care-takers of case-patients who were informed that their participation was voluntary and their refusal would not result in adverse consequences. All case-patients identified in the community were referred for free treatment at the cholera treatment centers. To ensure confidentiality of the respondents, each case-patient, case-household, and control household was assigned a unique identifier which was used instead of their names during data analysis.

## Results

### Descriptive epidemiology and hypothesis generation findings

**Fig. 2:**
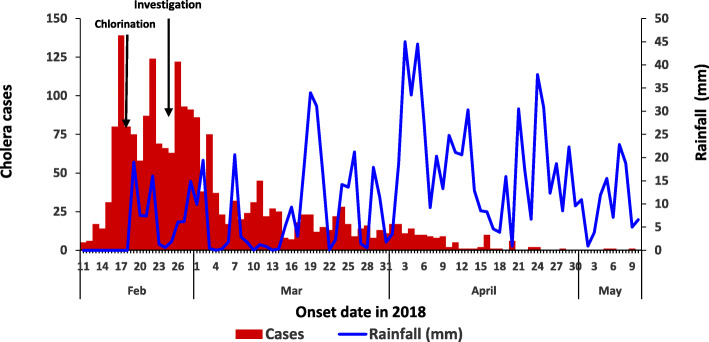
Cholera cases by date of onset and rainfall in Hoima District: February to May 2018

**Fig. 3:**
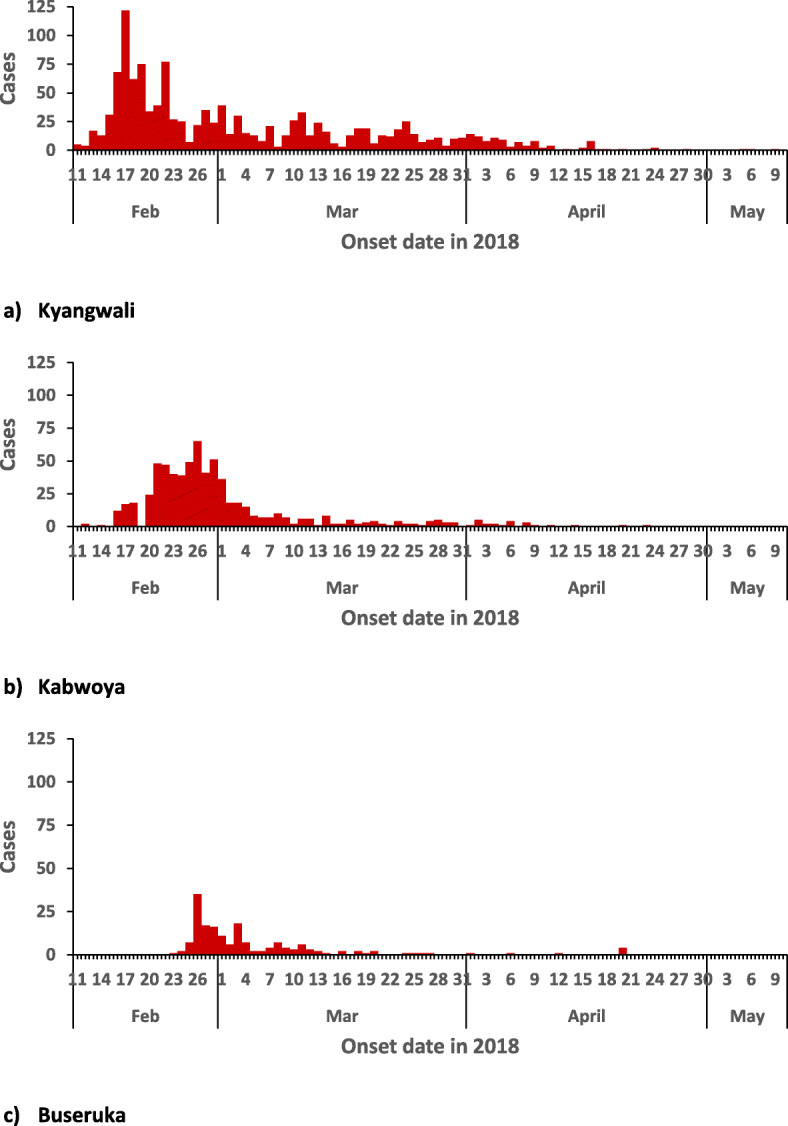
Cholera outbreak stratified by sub-county in Hoima District: February–May, 2018. **a–c** Cholera outbreak stratified by sub-county in Hoima District: February–May, 2018. Note. Three counties with a total of 51 cases are not shown

We identified 2122 case-patients and 44 deaths (CFR = 2.1%). The commonest signs in case-patients were acute watery diarrhea (100%) and vomiting (90%). The overall attack rate in Hoima District was 3.2/1000, with Kyangwali sub-county being the most affected (attack rate [AR] = 13/1000), followed by Kabwoya (AR = 10/1000), Buseruka (AR = 4.0/1000), Kahoora Division (AR = 0.31/1000), Bugambe (AR = 0.27/1000), and Bujumbura Division (AR = 0.090/1000) (Fig. 1). Children aged 2–5 years were the most affected age group (AR = 5.3/1000) (all children ≤ 5 years was used as denominator, as census data were not available for children 2–5 years) (Table 1). Persons originating from DRC were affected at an attack rate 10 times greater than persons from Uganda (AR = 15/1000 vs 1.4/1000) (Table 1). The outbreak lasted for 4 months. Multiple heavy rainfalls occurred in between several peaks of the outbreak (Fig. 2). A retrospective review of health records in the affected area (refugee settlement) on 16 March 2018 revealed that the index case was seen on 11 February 2018 at one of the health facilities in the refugee settlement area. The index case was an 11-year-old recently-arrived male refugee from the DRC who presented with watery diarrhea and vomiting. A cholera outbreak had been ongoing in areas near his residence in the DRC at the time of his departure [26, 27]. From 11 February, there was a rapid increase in the number of cases, and subsequent waxing and waning of the outbreak, with peaks on 17, 22, and 26 February. Stratification of the epi-curve by sub-county demonstrated that the outbreak started in Kyangwali before spreading to other sub-counties (Figs. 1 and 3). Ten (71%) of the 14 case-patients interviewed reported drinking stream water; three (21%) drank tap water, and one (7%) drank spring water. For this reason, stream water was identified as a potential risk factor.

**Table 1:** Attack rates (AR) by gender, nationality, and age during cholera outbreak, Hoima District, Uganda: February–May, 2018

Demographic feature	***n***	%	Population	AR/1000
**Gender**				
Female	1055	49.7	330 800	3.2
Male	1067	50.3	338 100	3.2
**Nationality**				
Ugandan	846	40	585 900	1.4
DRC	1276	60	83 000	15
**Age, years**				
2–5^a^	764	36	143 930	5.3
6–17	580	27	203 430	2.9
18–30	420	20	157 620	2.7
31–59	274	13	136 520	2.0
60–95	84	4	27 400	3.1

^a^Denominator used was children 0–5 years due to unavailability of data for children aged 2–5 years

*DRC-* Demographic Republic of Congo

### Case-control study findings

We enrolled 73 case-households and 107 control-households in the study. To explore opportunities for interaction or confounding among different water sources, we computed odds ratios for illness among persons who reported drinking all possible combinations of the three water sources (Table 2).

**Table 2:** Association of illness with reported water source during cholera outbreak, Hoima District, Uganda: February–May, 2018

Water Drunk	Percent of		***OR***	95% ***CI***
	Cases, *n* = 73	Controls, *n* = 107		
No spring, tank, or stream^a^	1.4	15	Reference.	–
Spring only	0	0.93	0	–
Tank only	33	31	11.6	1.4–94
Stream only	11	8.4	14.2	1.5–133
Spring and tank only	0	10	0	–
Spring and stream only	0	0.93	0	–
Tank and stream only	53	34	17.3	2.2–137
Stream and tank and spring	1.4	0	Infinity.	–

^a^Water source was likely collected rainwater. *OR* Odds ratio; 95% *CI* Confidence interval (The probability that a population parameter will fall between the two set values for 95% of the times); − not applicable

Seventeen (9.4%) of the 180 persons did not report drinking any of the three water types about which we inquired (these people likely drank collected rain water, but we did not inquire about this water source). Using this group as a reference, stream water appeared to be the single source most strongly associated with illness (*OR* = 14.2; 95% *CI*: 1.5–133), although tank water also appeared to be independently associated with illness (*OR* = 11.6; 95% *CI*: 1.4–94). Persons who drank tank and stream water had a 17-fold higher odds of illness compared with persons who drank none of the three types of water (*OR* = 17.3, 95% *CI*: 2.2–137). Spring water was not independently associated with illness; it was consumed by only one case-patient, who also reported consuming stream water and tank water.

### Environmental assessment and laboratory findings

The most affected villages in Kyangwali sub-county were Maratatu B and Maratatu C. Maratatu B and C were located on either side of the valley separated by the stream. Households in Maratatu B and C were settled as close as two metres from the stream. It took more than 2 h to access the tank water due to the long queues, while it took less than 10 min to access spring water and less than 5 min to access stream water. There was evidence of open defecation along the stream. Water testing results revealed none of the water samples from the tank or the spring had any coliforms, while all six water samples from the stream had > 100 fecal coliforms each. We observed one latrine in Maratatu C and none in Maratatu B. All 130 stool samples from suspected cholera cases tested positive for cholera by RDT, and 124 (95%) tested positive by culture. All the culture-confirmed samples were collected from 16 February to 9 May, 2018. Stream water was shared between the refugees and local residents while tank water and spring water was not shared.

## Discussion

We identified a cholera outbreak in Hoima District, Uganda, during February–May 2018. This was one of the largest cholera outbreak in Uganda since 2012, affecting > 2000 persons, most of whom were refugees from the Democratic Republic of Congo. Both stream water and tank water sources were found to be independently associated with illness. Stream water was found to be highly contaminated with fecal matter, rendering it unsafe to drink.

This was the fifth cholera outbreak in Hoima District and the third in Kyangwali sub-county since 2012 [14–17]. Kyangwali, the most affected area, is a refugee-hosting community, primarily for refugees coming from the DRC. The DRC was struggling with a cholera outbreak at the time and it is likely that the outbreak was introduced from cross-border travel of refugees [28]. Other studies in Uganda, including molecular characterization studies, have also noted cholera importation risks from cross-border travel and influxes of refugees during conflicts [29, 30].

Our environmental findings revealed evidence of open defecation along the stream in Kyangwali refugee settlement. Open defecation near drinking water sources has been associated with cholera outbreaks in the past [31–34]. In Kasese District, Uganda, a cholera outbreak was also associated with open defecation along the streams [35]. Defecation by the stream was possibly due to shortage of latrines. This was evidenced by our environmental findings where only one latrine (with two stances) was available for two blocks (covering approximately 4000 persons). Increased access to latrines in the settlement and sensitization of the communities about latrine usage is warranted.

The role of tank water in the outbreak is unclear. Tank water was obtained from the nearby Lake Albert and transported using a water truck. The water was supposed to have been chlorinated before the outbreak; however, it is unclear whether or not this occurred. There was no evidence of fecal contamination in tank water at the time of our investigation. However, by the time we began our investigation, interventions – including chlorination of the water tanks – had already been instituted to ensure safe water was being provided. It is possible that tank water was initially contaminated, and subsequently treated to render it safe, leading to an association with illness without evidence of contamination at the time of sampling. The initial contamination of tank water was because tank water was drawn from feacally contaminated Lake Albert. Lake Albert was probably contaminated by feacally contaminated rivers that flowed into the Lake, or direct defecation into the Lake by the communities due to lack of latrines. As good public health practice, steps should be taken to ensure that chlorination of lake-sourced tank water continues.

Repeated heavy rainfalls in the area might have exacerbated the outbreak. Several heavy rainfalls were followed by peaks in cases a few days later. Other studies have also noted this pattern [36, 37]. Heavy rainfall can wash fecal matter into water sources – such as from stream shores into the stream itself – and thereby contaminate it. This underscores the importance of increasing population access to latrines in this area, particularly during rainy seasons. In addition, when the outbreak was declared and public health action started, people were advised to drink only treated water leading to fewer new cases. However, to prevent exacerbation of cholera cases, there should also be continuous education of the communities about the significance of treating their drinking water, either by boiling or chlorination. In this location, chlorination of drinking water is a more feasible option to many community members, as chlorination tablets are readily available and affordable in village shops.

The proximity of Western Uganda to active cholera hotspots in the DRC as well as the continuous influx of refugees from DRC to Uganda creates a highly vulnerable area for recurrent cholera outbreaks [38]. Patients with cholera can easily move across Lake Albert to Hoima District. Improved surveillance along Lake Albert and screening of all persons arriving at lake landing sites from DRC might reduce the frequency of outbreaks. However, even screening may not prevent such outbreaks. While ill persons may have crossed the border and spread the infection (data indicates that index cases presented with diarrhea and vomiting), as many as 75% of persons with cholera are asymptomatic or have only mild diarrhoea, yet still shed the bacteria in their stool [39, 40]. Alternately, infected persons might have crossed the border while incubating the disease; the incubation period for cholera can be as long as 5 days [41]. In high-risk areas such as the villages along the border, consideration should be given to conducting a two-dose cholera vaccine campaign. This would protect recipients against cholera for 3 to 5 years, with approximately 80% effectiveness [42]. Additional interventions also need to focus on secondary prevention. A permanent supply of safe water in Kyangwali Refugee Centre through construction of boreholes, or continuous supply of treated water from the nearby lake, could reduce the incidence of outbreaks of waterborne diseases in this area. Without such interventions and with continued refugee influxes, these outbreaks are likely to continue.

This study had some limitations. Given that the index case occurred on 11 February, 2018 and we went in for the investigation 2 weeks later, we could have missed some cholera cases in the communities. Failure to account for all cholera cases in the communities may have resulted in an underestimation of the scope of the outbreak.

Hoima District Health Team in conjunction with Ministry of Health, Red Cross and Medical Teams International provided case-patients with oral rehydration salts and transported critically ill patients to designated cholera treatment centers for rehydration and treatment with antibiotics. United Nations High Commission for Refugees provided chlorinated water and temporary latrines to the refugee community in Kyangwali Refugee Settlement. The Ministry of Health, in conjunction with partners, conducted widespread education on local radio and television stations, and direct community engagements about personal hygiene, early reporting of suspected cases, general sanitation, and cholera control measures. From 2 to 6 May 2018, and again from 6 to 10 June 2018, the Ministry of Health, with support from World Health Organization, vaccinated at least 360 000 people against cholera in the hotspot areas of Hoima District. This was a two-dose vaccination campaign (i.e same people were vaccinated twice).

## Conclusions

Our investigation demonstrated that this was a prolonged cholera outbreak that affected four sub-counties and two divisions in Hoima District and was associated with drinking of contaminated stream water. We recommended to Hoima District and Ministry of Health that the interventions around safe water, chlorination of drinking water, increased latrine coverage, and sensitization of the communities about basic cholera control measures be continued to reduce the risk of the next cholera outbreak.

## Data Availability

The datasets upon which our findings are based belong to the Uganda Public Health Fellowship Program. For confidentiality reasons, the datasets are not publicly available. However, the data sets can be availed upon reasonable request from the corresponding author and with permission from the Uganda Public Health Fellowship Program.

## Abbreviations

AR: Attack rate
US CDC: US Centers for Disease Control and Prevention
CFR: Case fatality rate
CFU: Coliform forming unit
*CI*: Confidence interval
CTC: Cholera Treatment Centre
DRC: Demographic Republic of Congo
IDI: Infectious Diseases Institute
MoH: Ministry of Health
MPN: Most Probable Number
MTI: Medical Team International
*OR*: Odds ratio
PHFP: Public Health Fellowship Program
RDT: Rapid diagnostic test
UNHCR: United Nations High Commission for Refugees
UNHLS: Uganda National Health Laboratory Services
UNICEF: United Nations International Children’s Emergency Fund
WFP: World Food Program
WHO: World Health Organization
